# Turning the Curve on Obesity Prevalence Among Fifth Graders in the Los Angeles Unified School District, 2001–2013

**DOI:** 10.5888/pcd14.160377

**Published:** 2017-02-16

**Authors:** Amanda Kamali, Heena Hameed, Margaret Shih, Paul Simon

**Affiliations:** 1 Epidemic Intelligence Service, Centers for Disease Control and Prevention, Los Angeles County Department of Public Health, Acute Communicable Disease Control, Los Angeles, California; 2 Los Angeles County Department of Public Health, Office of Health Assessment and Epidemiology, Los Angeles, California; 3 Los Angeles County Department of Public Health, Division of Chronic Disease and Injury Prevention, Los Angeles, California

## Abstract

**Introduction:**

After multiple decades of increasing childhood obesity prevalence in the United States, findings from recent studies suggest that prevalence has leveled or is decreasing in some populations. However, demographic and socioeconomic disparities in prevalence remain and may be increasing.

**Methods:**

To assess recent trends and disparities in childhood obesity prevalence in Los Angeles County, we analyzed data from 2001 through 2013 in fifth graders in the Los Angeles Unified School District (LAUSD). Obesity was defined as a body mass index at or above the 95th percentile for children of the same age and sex as compared with Centers for Disease Control and Prevention growth charts, on the basis of measured height and weight. Trends were examined by sex, race/ethnicity, and socioeconomic status (SES). SES was determined by using school-level data on the percentage of students participating in a free and reduced-price meal program.

**Results:**

Obesity prevalence increased from 27.5% in 2001 to 31.6% in 2005, was stable from 2005 through 2010, and decreased from 31.6% in 2010 to 28.5% in 2013. Similar trajectories in prevalence were observed for all demographic and SES subgroups, although the decline in prevalence began earlier among whites and students attending schools in the highest SES group. Disparities in prevalence by race/ethnicity and SES were observed during the entire study period but narrowed slightly from 2010 through 2013.

**Conclusion:**

Although obesity prevalence among fifth graders in LAUSD declined from 2010 through 2013, prevalence remains higher than in 2001, and demographic and socioeconomic disparities in prevalence persist. Future interventions in the county should prioritize Latinos and students attending low SES schools.

## Introduction

From the late 1970s to 2000, the prevalence of childhood obesity rose rapidly throughout the United States (1). Since then, obesity prevalence has increased at a considerably slower rate among 12- to 19-year-olds and has begun to decline among 6- to 11-year-olds and 2- to 5-year-olds nationally (2). Recent declines in childhood obesity prevalence have also been reported in certain states (3–5), a regional population (6), and several large cities (7–9). Despite these promising trends, racial/ethnic and socioeconomic disparities in childhood obesity prevalence remain and may be increasing (3,5,10).

The Los Angeles Unified School District (LAUSD) is the second-largest school district in the United States, with more than 500,000 students enrolled during 2013 to 2014. The district also includes the largest Latino student population in the nation, and approximately 80% of all students are eligible for participation in LAUSD’s free and reduced-price meal (FRPM) program. From 2001 through 2013, childhood obesity prevention programs and policy efforts expanded in Los Angeles County. This expansion was accelerated in 2010 with funding from the Centers for Disease Control and Prevention’s (CDC’s) Communities Putting Prevention to Work (CPPW) initiative and CDC’s Community Transformation Grant (CTG) program. We aimed to describe the changing prevalence of obesity and demographic and socioeconomic disparities in prevalence among fifth graders in LAUSD during this period. This group has the highest prevalence of obesity reported among children and adolescents in Los Angeles County (11).

## Methods

We analyzed fifth-grade student data collected during the spring from 2001 through 2013 via the California Physical Fitness Testing Program. The program requires annual fitness testing of all fifth, seventh, and ninth graders in all public schools, including measures of body composition, aerobic capacity, strength, and flexibility. In LAUSD, body composition is assessed by measured height and weight. Teachers or trained staff perform the height and weight measurements using standardized procedures, including removal of shoes and rounding down to the nearest inch for height and nearest pound for weight (12,13). Measurements are reported by the school district to the California Department of Education (CDE). Demographic information about each student, including age (in years), sex, and race/ethnicity, is also reported. Data for 2001 through 2010 were obtained from CDE and data for 2011, 2012, and 2013 were obtained from LAUSD.

In the absence of student-level data about socioeconomic status (SES), we used, as a proxy, state data that specified percentage of students enrolled or eligible to participate in the FRPM program at each school during the fall from 2000 through 2012 (14,15). Students attending schools with more than 75% of students enrolled in the program were classified as low SES, those attending schools with 51% to 75% enrolled were classified as middle SES, and those attending schools with 50% or less enrolled were classified as high SES. The percentage of schools in each SES group was consistent throughout the study period except in 2008, when the number of schools in the lowest SES group decreased by 15%, the number in the middle group increased by 24%, and the number in the highest group increased by 42%. To address potential misclassification, we used an algorithm to reclassify some schools in 2008. If a school had the same SES classification throughout the study period except in 2008, we reassigned it that classification for 2008. This resulted in reclassification of 13% of schools in 2008.

We calculated age- and sex-specific body mass index (BMI) percentiles on the basis of measured height and weight and classified students as obese (≥95th percentile for age and sex) using CDC growth charts (16). For the entire study period, we excluded data from the school health record with missing values (3.6% of students) as well as height, weight, and calculated BMI values that were biologically implausible on the basis of CDC standards (0.7% of students) (17).

Trends in obesity prevalence were assessed by sex, race/ethnicity, and school SES. Racial/ethnic trends included whites, blacks, and Latinos. The trend for Asian students was not calculated because of concern about misclassification; the number of students classified as Asian was considerably lower than the number of Asian students based on enrollment statistics. Trends for American Indian or Alaska Native and Native Hawaiian or Other Pacific Islander students could not be calculated because CDE did not provide their racial/ethnic information to protect student confidentiality. Statistical analysis was performed by using the Cochran–Armitage test for trend (18,19). All analyses were completed by using SAS version 9.3 (SAS Institute Inc).

The study was reviewed by CDC for the purpose of human subjects’ protection and deemed to be nonresearch. It was also reviewed by the Los Angeles County Department of Public Health institutional review board and certified as exempt.

## Results

The number of fifth-grade students included in this analysis decreased from 56,363 in 2001 to 44,181 in 2013, paralleling a similar decline in fifth-grade enrollment during this period in LAUSD. The percentage of enrolled students included in our analysis each year (ie, reporting completeness) ranged from a low of 86% in 2010 to a high of 98% in 2006. A small degree of variation in the demographic and socioeconomic distributions of students was observed during the study period (Table 1). The percentage of students that were male ranged from 50.2% to 51.2%, the percentage that were Latino ranged from 70.5% to 81.6%, the percentage that were black ranged from 7.3% to 12.6%, and the percentage that were white range from 7.0% to 10.3%. The percentage of students that attended low-SES schools ranged from 71.6% to 79.7%.

**Table 1:** Demographic and Socioeconomic Characteristics of Fifth-Grade Students, Los Angeles Unified School District, California, 2001–2013

Year	No.^a^>	Sex, %		Race/Ethnicity, %				School SES, %			
		Male	Female	White	Latino	Black	Other/ Unknown	Lowest^b^	Middle^c^	Highest^d^	Unknown^e^
2001	56,363	50.8	49.2	10.3	70.5	12.6	6.6	75.8	12.2	11.8	0.2
2002	57,883	50.2	49.8	9.6	72.0	12.2	6.3	77.4	11.7	10.9	0.0
2003	53,270	50.5	49.5	9.9	71.4	12.0	6.7	76.3	11.8	11.2	0.7
2004	56,726	51.1	48.9	8.6	73.7	11.4	6.4	79.5	10.6	9.9	0.1
2005	58,110	50.9	49.1	8.4	74.2	10.9	6.5	78.6	11.0	10.2	0.1
2006	56,566	51.2	48.8	8.5	74.3	10.8	6.5	79.5	9.5	10.9	0.1
2007	53,012	51.1	48.9	8.8	73.6	10.7	6.9	76.6	11.5	11.5	0.4
2008	49,453	51.2	48.8	7.1	76.2	7.4	9.3	75.7	12.0	11.9	0.4
2009	48,001	51.1	48.9	7.0	75.8	7.3	10.0	75.9	11.5	12.4	0.3
2010	43,919	51.1	48.9	7.9	81.6	7.5	2.9	79.7	9.8	10.2	0.2
2011	47,648	51.0	49.0	9.4	73.9	9.8	6.9	72.4	14.3	13.0	0.3
2012	45,567	50.8	49.2	9.6	74.1	9.6	6.7	74.2	14.1	11.6	0.2
2013	44,181	51.2	48.8	9.9	73.6	9.4	7.1	71.6	14.0	14.4	0.0

Abbreviation: SES, socioeconomic status.

a Students with valid body mass index information obtained from physical fitness testing.

b Students attending schools with >75% of students enrolled in free and reduced price meal program.

c Students attending schools with 51%–75% of students enrolled in free and reduced price meal program.

d Students attending schools with ≤50% of students enrolled in free and reduced price meal program.

e Students attending schools with missing information about free and reduced price meal program participation.

The prevalence of obesity among fifth graders increased from 27.5% in 2001 to 31.6% in 2005 (*P* < .001), was stable from 2005 through 2010 (*P* = .78), and decreased from 31.6% in 2010 to 28.5% in 2013 (*P* < .001) (Figure 1A). Obesity prevalence was higher among males than females throughout the study period, but the temporal trend in prevalence was similar for both groups (Figure 1B). However, the sex disparity in prevalence varied by race/ethnicity (Figure 1C). The prevalence was higher for whites and Latinos among males than females, but among blacks the prevalence was higher among females than males. The highest prevalence was observed among Latino boys (39.6% in 2005).

**Figure 1A:**
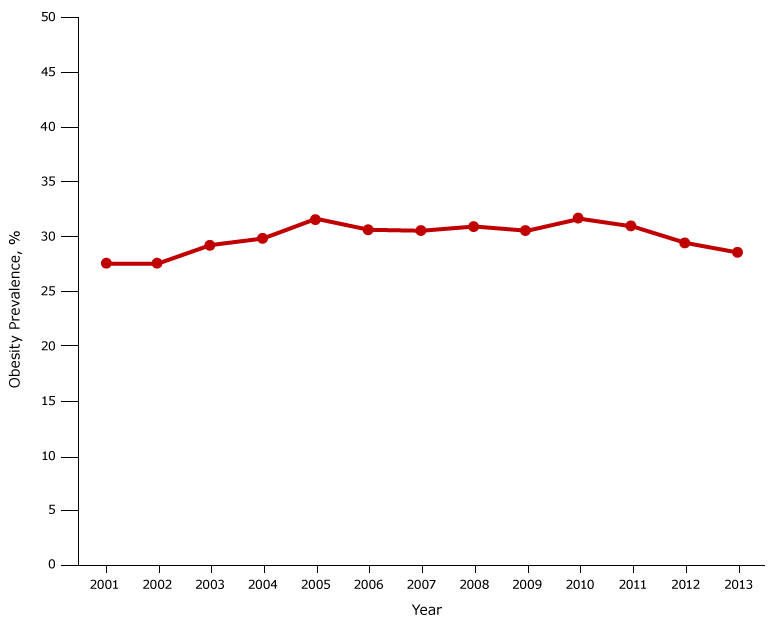
Obesity prevalence among fifth-grade students, Los Angeles Unified School District, California, 2001–2013. From 2001 through 2005, obesity prevalence increased from 27.5% to 31.6%. From 2010 through 2013, prevalence declined from 31.6% to 28.5%. **Table:** 

Year	Percentage
2001	27.5
2002	27.5
2003	29.2
2004	29.8
2005	31.6
2006	30.6
2007	30.5
2008	30.9
2009	30.5
2010	31.6
2011	30.9
2012	29.4
2013	28.5

**Figure 1B:**
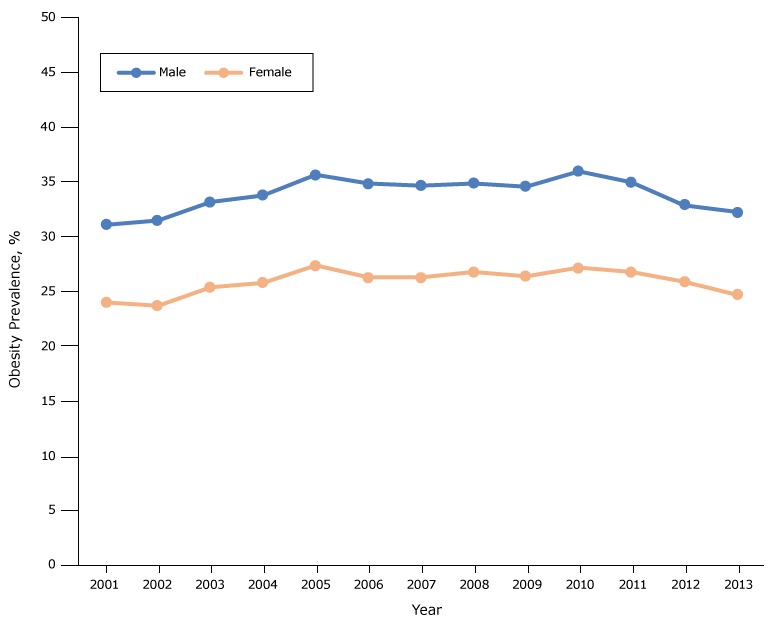
Obesity prevalence among fifth-grade students, by sex, Los Angeles Unified School District, California, 2001–2013. Throughout the study period, obesity prevalence was higher among males than among females. **Table:** 

Year	Percentage	
	Male	Female
2001	31.0	23.9
2002	31.4	23.6
2003	33.1	25.3
2004	33.7	25.7
2005	35.6	27.3
2006	34.8	26.2
2007	34.6	26.2
2008	34.8	26.7
2009	34.5	26.3
2010	35.9	27.1
2011	34.9	26.7
2012	32.8	25.8
2013	32.2	24.6

**Figure 1C:**
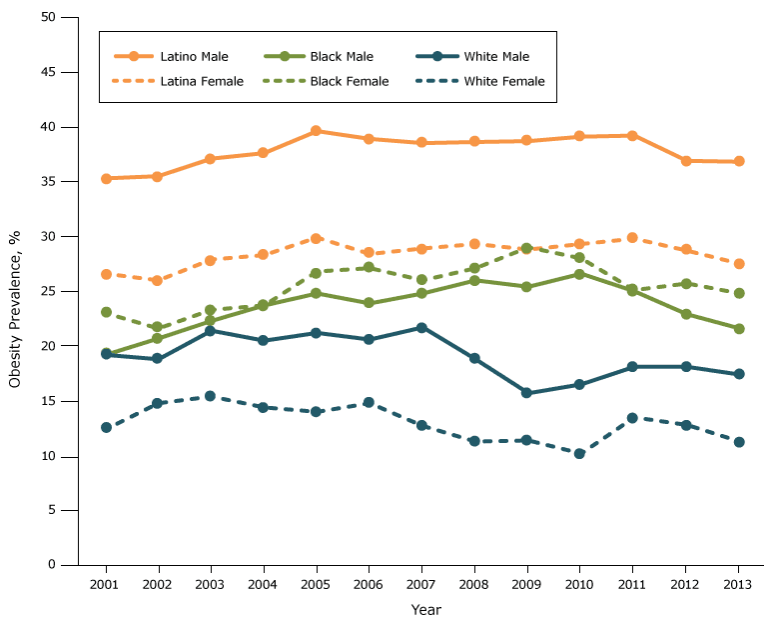
Obesity prevalence among fifth-grade students, by sex and race/ethnicity, Los Angeles Unified School District, California, 2001–2013. Obesity prevalence was higher among males than females for whites and Latinos, but for blacks the prevalence was higher among females than males. **Table:** 

Year	Race/Ethnicity, %					
	Male			Female		
	White	Latino	Black	White	Latina	Black
2001	19.2	35.3	19.2	12.4	26.6	23.0
2002	18.8	35.5	20.7	14.8	26.0	21.6
2003	21.4	37.1	22.3	15.4	27.9	23.3
2004	20.5	37.6	23.7	14.4	28.3	23.7
2005	21.2	39.6	24.8	14.0	29.9	26.8
2006	20.6	38.9	23.9	14.8	28.4	27.1
2007	21.7	38.5	24.8	12.7	28.9	26.0
2008	18.8	38.6	26.0	11.3	29.3	27.1
2009	15.7	38.7	25.4	11.4	28.8	29.0
2010	16.5	39.1	26.6	10.2	29.3	28.0
2011	18.1	39.2	25.0	13.5	29.8	25.1
2012	18.1	36.9	22.9	12.8	28.7	25.7
2013	17.4	36.8	21.6	11.3	27.5	24.8

For race/ethnicity among males and females combined, obesity prevalence throughout the study period was highest among Latinos, intermediate among blacks, and lowest among whites (Figure 1D). After initial increases in prevalence among these 3 groups, prevalence declined, starting in 2003 among whites, 2010 among blacks, and 2011 among Latinos.

**Figure 1D:**
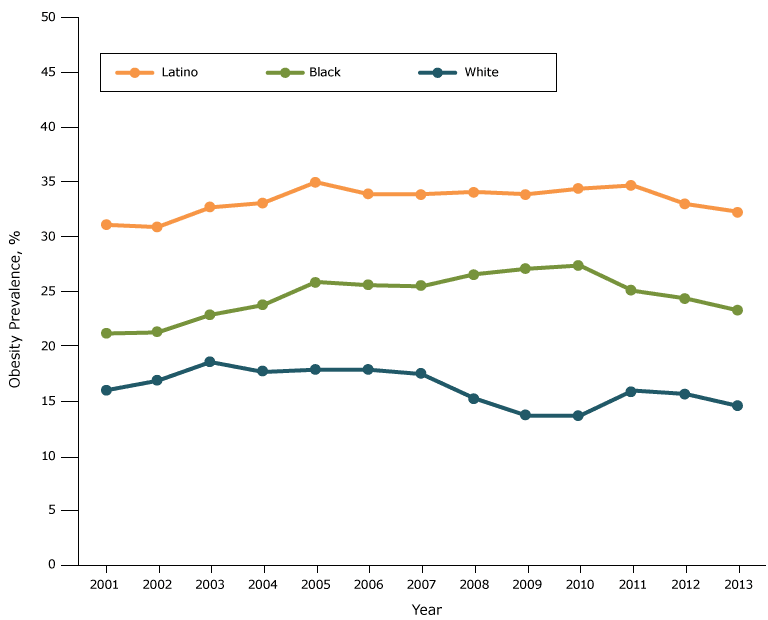
Obesity prevalence among fifth-grade students, by race/ethnicity, Los Angeles Unified School District, California, 2001–2013. Throughout the study period, obesity prevalence was lowest among white students, second lowest among black students, and highest among Latino students. **Table:** 

Year	Race/Ethnicity, %		
	White	Latino	Black
2001	15.9	31.0	21.1
2002	16.8	30.8	21.2
2003	18.5	32.6	22.8
2004	17.6	33.0	23.7
2005	17.8	34.9	25.8
2006	17.8	33.8	25.5
2007	17.4	33.8	25.4
2008	15.2	34.0	26.5
2009	13.6	33.8	27.0
2010	13.6	34.3	27.3
2011	15.9	34.6	25.0
2012	15.6	32.9	24.3
2013	14.5	32.2	23.2

For SES, obesity prevalence was highest among students in schools in the low-SES group, intermediate among those in the middle-SES group, and lowest among those in the high-SES group (Figure 2A). After initial increases in prevalence in all 3 groups, prevalence declined, starting in 2003 for those at schools in the high-SES group, 2008 for those at schools in the middle-SES group, and 2010 for those at schools in the low-SES group.

**Figure 2A:**
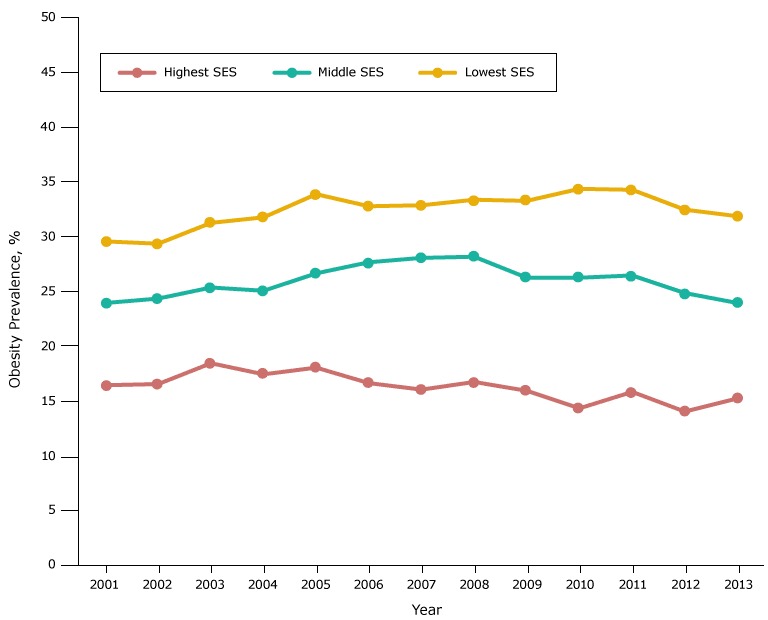
Obesity prevalence among fifth-grade students, by socioeconomic status (SES), Los Angeles Unified School District, California, 2001–2013. Throughout the study period, obesity prevalence was lowest among students in the high-SES group and highest among students in the low-SES group. **Table:** 

Year	Socioeconomic Status, %		
	Lowest	Middle	Highest
2001	29.7	24.1	16.6
2002	29.5	24.5	16.7
2003	31.4	25.5	18.6
2004	31.9	25.2	17.6
2005	34.0	26.8	18.2
2006	32.9	27.8	16.8
2007	33.0	28.2	16.2
2008	33.5	28.3	16.9
2009	33.4	26.4	16.1
2010	34.5	26.4	14.5
2011	34.4	26.6	16.0
2012	32.6	25.0	14.2
2013	32.0	24.1	15.4

In each racial/ethnic group, the obesity prevalence in most years was highest in the low-SES group, intermediate in the middle-SES group, and lowest in the high-SES group (Figures 2B–D). However, the gradient in prevalence across SES groups was largest among whites.

**Figure 2B:**
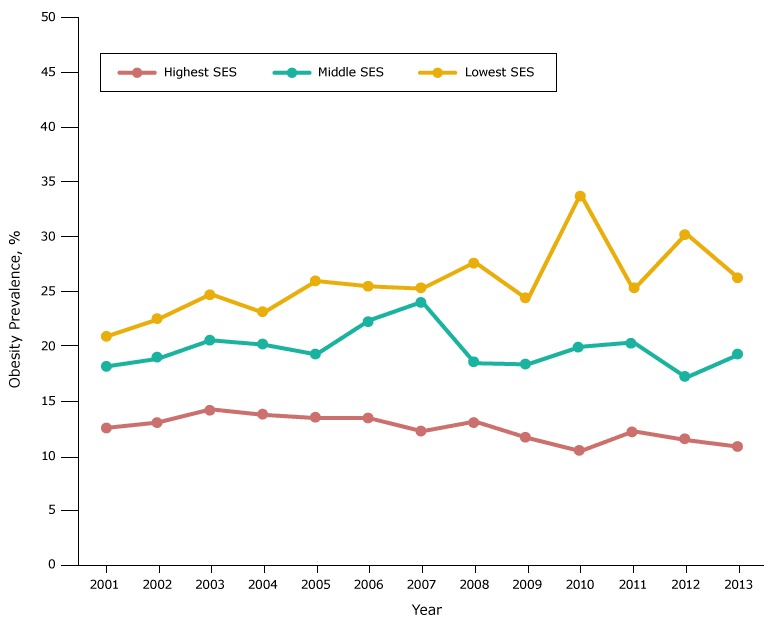
Obesity prevalence among white fifth-grade students, by socioeconomic status (SES), Los Angeles Unified School District, California, 2001–2013. Throughout the study period, obesity prevalence was lowest among white students in the high-SES group and highest among white students in the low-SES group. Obesity prevalence in 2008 for white students in the low SES group should be interpreted cautiously because of the limited number of students with body mass index information. **Table:** 

Year	Socioeconomic Status, %		
	Lowest	Middle	Highest
2001	21.0	18.3	12.7
2002	22.6	19.0	13.2
2003	24.9	20.7	14.4
2004	23.2	20.3	13.9
2005	26.1	19.4	13.6
2006	25.6	22.5	13.6
2007	25.4	24.2	12.4
2008	27.8	18.6	13.3
2009	24.4	18.5	11.8
2010	34.0	20.1	10.6
2011	25.3	20.5	12.4
2012	30.4	17.3	11.6
2013	26.3	19.4	11.0

**Figure 2C:**
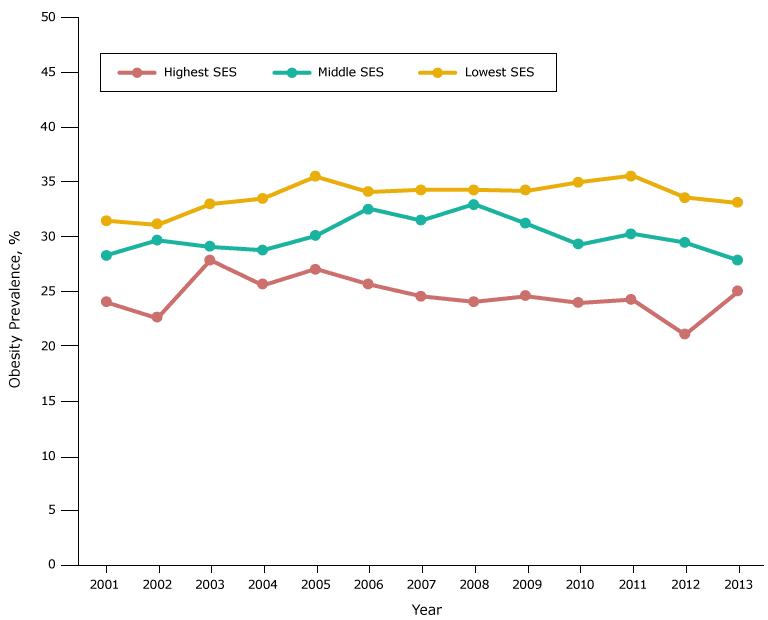
Obesity prevalence among Latino fifth-grade students, by socioeconomic status (SES), Los Angeles Unified School District, California, 2001–2013. Throughout the study period, obesity prevalence was lowest among Latino students in the high-SES group and highest among Latino students in the low-SES group. **Table:** 

Year	Socioeconomic Status, %		
	Lowest	Middle	Highest
2001	31.6	28.4	24.2
2002	31.2	29.8	22.7
2003	33.1	29.2	28.0
2004	33.6	28.9	25.7
2005	35.6	30.2	27.2
2006	34.2	32.7	25.8
2007	34.4	31.6	24.7
2008	34.4	33.1	24.2
2009	34.3	31.3	24.7
2010	35.1	29.4	24.1
2011	35.7	30.4	24.4
2012	33.7	29.6	21.2
2013	33.2	28.0	25.1

**Figure 2D:**
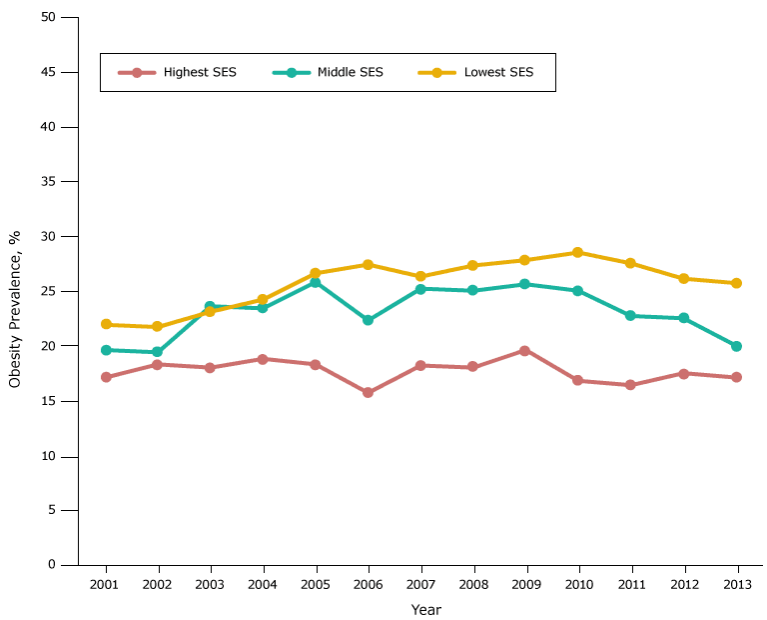
Obesity prevalence among black fifth-grade students, by socioeconomic status (SES), Los Angeles Unified School District, California, 2001–2013. Throughout most years of the study period, obesity prevalence was lowest among black students in the high-SES group, and highest among black students in the low-SES group. Obesity prevalence from 2008 through 2010 for black students in the high-SES group should be interpreted cautiously because of the limited number of students with body mass index information. **Table:** 

Year	Socioeconomic Status, %		
	Lowest	Middle	Highest
2001	22.1	19.8	17.3
2002	21.9	19.6	18.5
2003	23.3	23.8	18.2
2004	24.4	23.6	19.0
2005	26.8	26.0	18.5
2006	27.6	22.5	15.9
2007	26.5	25.4	18.4
2008	27.5	25.2	18.2
2009	28.0	25.8	19.8
2010	28.7	25.2	17.0
2011	27.7	22.9	16.6
2012	26.3	22.7	17.7
2013	25.9	20.2	17.3

During the most recent period for which data were available, 2010 through 2013, obesity prevalence declined among fifth graders overall, and a downward trend in prevalence was observed among Latino students (*P* < .001) and black students (*P* < .001) but not among white students. The decline in prevalence in each racial/ethnic group was greater among children in schools in the low-SES and middle-SES groups than in the high-SES group (Table 2). For children at schools in the low-SES group, obesity prevalence was highest among Latinos (33.2% in 2013) and similar among whites (26.3%) and blacks (25.9%). Among children in the high-SES group, larger disparities in prevalence were observed by race/ethnicity (25.1%, 17.3%, and 11.0% among Latinos, blacks, and whites, respectively, in 2013).

**Table 2:** Obesity^a^ Prevalence Among Fifth-Grade Students, by Race/Ethnicity and Socioeconomic Status, Los Angeles Unified School District, California, 2010–2013

Characteristic/SES Status	Year, %				Relative % Change	*P* Value^b^
	2010	2011	2012	2013		
**White**	13.6	15.9	15.6	14.5	6.6	.53
Lowest^c^	34.0	25.3	30.4	26.3	−22.6	.42
Middle^d^	20.1	20.5	17.3	19.4	−3.5	.38
Highest^e^	10.6	12.4	11.6	11.0	3.8	.92
**Latino**	34.3	34.6	32.9	32.2	−6.1	<.001
Lowest^c^	35.1	35.7	33.7	33.2	−5.4	<.001
Middle^d^	29.4	30.4	29.6	28.0	−4.8	.10
Highest^e^	24.1	24.4	21.2	25.1	4.1	.93
**Black**	27.3	25.0	24.3	23.2	−15.0	<.001
Lowest^c^	28.7	27.7	26.3	25.9	−9.8	.01
Middle^d^	25.2	22.9	22.7	20.2	−19.8	.02
Highest^d^	17.0^f^	16.6	17.7	17.3	1.8	.75

Abbreviation: SES, socioeconomic status.

a Obesity was defined as a body mass index ≥95th percentile, according to Centers for Disease Control and Prevention growth charts (17).

b Calculated by using the Cochran-Armitage test for trend.

c Students attending schools with >75% of students enrolled in free and reduced price meal program.

d Students attending schools with 51%–75% of students enrolled in free and reduced price meal program.

e Students attending schools with ≤50% of students enrolled in free and reduced price meal program.

f Estimate should be interpreted cautiously because of the limited number of students with body mass index information in this stratum.

## Discussion

From 2001 through 2013, an initial increase in obesity prevalence among fifth graders in LAUSD was followed by a leveling and then a decline in prevalence. A similar pattern was observed for all demographic and socioeconomic subgroups. The observed decline is consistent with reported declines among elementary school–aged children in New York City (8), Philadelphia (7), and the Kaiser Permanente health care system in southern California (10).

Despite this favorable trend, the obesity prevalence in our study population (28.5%) in 2013 is above the 2001 prevalence and is considerably higher than the prevalence reported nationally (17.5%) among 6- to 11-year-olds from 2011 through 2014 (2). We found a higher prevalence in males than females among whites and Latinos but not among blacks, a pattern that has also been reported statewide in California (5). Latino males had the highest prevalence of obesity in our study, a finding consistent with other studies, although the prevalence among Latino males reported here is far higher than has been reported elsewhere (2,5,7).

We also found an inverse relationship between obesity prevalence and SES in the total study group and in each racial/ethnic group. These disparities were wide during the entire study period but narrowed slightly from 2010 through 2013. The racial/ethnic disparities in prevalence were attenuated, although not eliminated, in low and middle socioeconomic strata, consistent with findings from other studies (20,21).

Although our study was not designed to evaluate the effects of local childhood obesity prevention interventions, changes in obesity prevalence trajectories occurred during a period of expanded programmatic and policy efforts. For example, LAUSD implemented a district-wide policy in 2004 that prohibited the sale of most sugar-sweetened beverages on school campuses (22). Efforts to improve the quality of physical education across the district were initiated in 2004 with technical assistance provided to schools through the establishment of a position for a central physical education advisor. In 2005, a state policy mandated nutrition standards for all competitive foods and beverages sold on school campuses (23). These and similar efforts have been associated with reductions in childhood obesity prevalence (24,25).

Local interventions were expanded in 2010 with a substantial infusion of funding through the federal CPPW initiative and continued in 2012 through CTG funding. For example, this funding supported LAUSD efforts to improve nutritional quality of school meals (26) and also helped leverage joint-use agreements to open school grounds for recreational purposes during nonschool hours (27). Additionally, intensive public education and media campaigns to reduce sugar-sweetened beverage consumption were associated with a modest decline in consumption among children in the county from 2007 through 2011 (28).

Our study has limitations. First, the analysis was limited to fifth graders in public school and may not be generalizable to other elementary school–aged children in Los Angeles County or in other jurisdictions. Second, we were unable to validate height and weight measurements in our data set; however, testing, including measurement of height and weight, was performed by staff trained in administering the fitness testing program. Third, our SES measures were determined on the basis of school-level enrollment in the FRPM program and may not accurately reflect the SES of individual students. For example, in high-income schools, Latino and black children may have been more likely than whites to live in low-income households. Lastly, because our study was a descriptive analysis, we could not formally assess the effect of specific programs or policies on obesity prevalence.

Despite these limitations, our findings suggest recent progress in addressing the childhood obesity epidemic in Los Angeles County. However, prevalence remains high, and substantial disparities in prevalence persist by sex and across racial/ethnic and socioeconomic subpopulations. Given the large size of the Latino child population in the county, further progress in reducing obesity prevalence will require more effective culturally tailored interventions. These interventions should extend beyond school environments to address community conditions, particularly conditions in low-income communities that promote unhealthy diets and physical inactivity. For example, efforts are under way in Los Angeles County to reduce the marketing of unhealthy food and beverages to children (29) and to expand access to affordable produce in neighborhood markets, including neighborhoods with large concentrations of Latino residents (30). However, these efforts are unlikely to achieve the desired population-level impacts unless implemented on a large scale.
